# High Intensity Resistance Exercise Training vs. High Intensity (Endurance) Interval Training to Fight Cardiometabolic Risk Factors in Overweight Men 30–50 Years Old

**DOI:** 10.3389/fspor.2020.00068

**Published:** 2020-06-16

**Authors:** Michael Tuttor, Simon von Stengel, Matthias Kohl, Michael Lell, Michael Scharf, Michael Uder, Andreas Wittke, Wolfgang Kemmler

**Affiliations:** ^1^Institute of Medical Physics, Friedrich-Alexander University Erlangen-Nürnberg, Erlangen, Germany; ^2^Department of Medical and Life Sciences, University of Furtwangen, Schwenningen, Germany; ^3^Institute of Radiology, University-Hospital Nürnberg, Nürnberg, Germany; ^4^Department of Radiology, University of Erlangen-Nürnberg, Erlangen, Germany

**Keywords:** high intensity interval training, high intensity resistance exercise training, single set resistance exercise training, cardiometabolic risk, cardiac parameters, metabolic syndrome

## Abstract

Cardiovascular and cardiometabolic diseases are leading causes of death worldwide. Exercise favorably affects this problem, however only few invest (enough) time to favorably influence cardiometabolic risk-factors and cardiac morphology/performance. Time-effective, high-intensity, low-volume exercise protocols might increase people's commitment to exercise. To date, most research has focused on high-intensity interval training (HIIT), the endurance type of HIT, while corresponding HIT-resistance training protocols (HIT-RT) are rarely evaluated. In this study we compared the effect of HIIT vs. HIT-RT, predominately on cardiometabolic and cardiac parameters in untrained, overweight-obese, middle-aged men. Eligible, untrained men aged 30–50 years old in full-time employment were extracted from two joint exercise studies that randomly assigned participants to a HIIT, HIT-RT or corresponding control group. HIIT predominately consisted of interval training 90 s−12 min, (2–4 sessions/week), HIT-RT (2–3 sessions/week) was applied as a single set resistance training to muscular failure. Core intervention length of both protocols was 16 weeks. Main inclusion criteria were overweight-obese status (BMI 25–35 kg/m^2^) and full employment (occupational working time: ≥38.5 h/week). Primary study-endpoint was the Metabolic Syndrome (MetS) Z-Score, secondary study-endpoints were ventricular stroke volume index (SVI) and myocardial mass index (MMI) as determined by Magnetic Resonance Imaging. The Intention to treat (ITT) principle was applied to analyze the summarized data set. Twenty-seven eligible men of the HIT-RT and 30 men of the HIIT group were included in the ITT. Both interventions significantly (*p* < 0.001) improve the MetS Z-Score, however the effect of HIIT was superior (*p* = 0.049). In parallel, HIT-RT and HIIT significantly affect SVI and MMI, with the effect of HIIT being much more pronounced (*p* < 0.001). Although HIIT endurance exercise was superior in favorably affecting cardiometabolic risk and particularly cardiac performance, both exercise methods positively affect cardiometabolic risk factors in this overweight to obese, middle-aged cohort of males with low time resources. Thus, the main practical application of our finding might be that in general overweight-obese people can freely choose their preferred exercise type (HIIT-END or HIT-RT) to improve their cardiometabolic health, while investing an amount of time that should be feasible for everybody.

**Trial Registrations:** NCT01406730, NCT01766791.

## Introduction

Cardiovascular and cardiometabolic diseases are leading causes of death worldwide (Rao, 2018). Physical activity and in particular exercise favorably affect the incidence and development of this problem (Börjesson et al., 2010; Lin et al., 2015), however only the minority of people (Clark, 1999; Rütten et al., 2005) exercise frequently enough to favorably affect cardiorespiratory and musculoskeletal fitness (Garber et al., 2011). Since most people gave time constraints as the main obstacle to exercising frequently (Rütten et al., 2009), time-effective exercise protocols might be appropriate methods for increasing people's compliance with exercise or training interventions. High intensity exercise training (HIT)—applied as either endurance (HIIT) or resistance exercise (HIT-RT) training—i.e., methods that feature low exercise volumes, are such candidates. While the effect of high intensity interval training (HIIT) on cardiovascular and cardiometabolic risk factors has been frequently addressed (e.g., Gibala, 2007; Kessler et al., 2012; Haykowsky et al., 2013; Weston et al., 2013, HIT-RT defined as single set exercise protocol with work to failure; Gießing, 2008; Steele et al., 2017a) has been rarely validated to the same extent. A recent study that applied HIT-RT reported significant effects on cardiometabolic risk factors including abdominal fat mass in 30–50 year-old men (Kemmler et al., 2016a). Few studies set out to address the effect of resistance exercise vs. endurance exercise programs on cardiometabolic risk factors in overweight-obese people (e.g., Bateman et al., 2011; Sigal et al., 2014; Ramirez-Velez et al., 2016). Data of the studies were inconsistent with comparable favorable effects on cardiometabolic markers (e.g., Sigal et al., 2014) or significantly higher effects of endurance vs. resistance exercise (e.g., Bateman et al., 2011). However, all the studies focus on time-consuming high volume/low intensity protocols, and only one non-realized study protocol (Ramirez-Velez et al., 2016) addressed middle-aged men, i.e., a cohort with particularly low time resources. This aspect is of importance since drop-out rates observed for the 4–6 month interventions of Bateman et al. (2011) and Sigal et al. (2014) were high (27 and 21%) and most participants reported time constraints and loss of interest as a reason for their withdrawal.

The aim of the present study was thus to compare the effects of two closely related exercise trials, one focusing on HIIT exercise, the other on HIT-RT, in an untrained cohort of overweight to obese men 30–50 years old with low time resources.

Our primary hypothesis was that (1) HIIT is significantly more effective for favorably affecting the Metabolic Syndrome Z-score (MetS Z-Score) compared with HIT-RT, while (2) both exercise protocols generated significant changes in the MetS Z-Score.

Our core secondary hypothesis was that HIIT is significantly more effective for favorably affecting (1) ventricular stroke volume index and (2) myocardial mass index compared with HIT-RT.

Another secondary hypothesis was that (1) HIIT is significantly more effective for favorably affecting body fat rate compared with HIT-RT while (2) HIT-RT is significantly more effective for favorably affecting Lean Body Mass (LBM) compared with HIIT.

## Materials and Methods

### Experimental Design

We compared two randomized controlled trials (Kemmler et al., 2015, 2016c) of comparable length with two independent cohorts of men 30–50 years old living in the area of Erlangen-Nürnberg. Both studies, i.e., the Running Strengthen the Heart (RUSH) study and the Physical Adaptions in Untrained on Strength and Heart (PUSH), were studies that focus on time-efficient exercise strategies applied with high intensity (HIT). In this paper we concentrate on the effect of high intensity interval training (HIIT) vs. high intensity resistance training (HIT-RT) on cardiometabolic risk factors and markers in a subgroup of overweight to obese men. The studies were initiated by the Institute of Medical Physics (IMP) and conducted in close cooperation with the Department of Radiology, Friedrich-Alexander University of Erlangen-Nürnberg (FAU). The studies were approved by the ethics committee of the FAU (RUSH: No. 4463, PUSH: No. 53_12 B) and complied with the Declaration of Helsinki “Ethical Principles for Medical Research Involving Human Subjects.” After detailed study information all participants gave written informed consent. The studies are fully registered under ClinicalTrials.gov (RUSH: NCT01406730, PUSH: NCT01766791).

### Participants

Briefly, we used the citizens' register of the municipality to contact 2,000 randomly selected men aged 30–50 years in the area of Erlangen-Nürnberg, Germany. Personalized letters gave detailed study information including the most relevant eligibility criteria [e.g., training status, study period, contraindications, Body Mass Index (BMI)]. Men who responded were checked for eligibility by phone and physical assessments before being invited to joint information sessions. Eligibility criteria applied for both studies were (1) untrained (i.e., ≤ one endurance (RUSH) or resistance (PUSH) exercise session/week; (2) pathological changes in the heart; (3) acute inflammatory diseases; (4) medication/diseases affecting cardiovascular system or muscles; (5) severe obesity (BMI >35 kg/m^2^); (6) contraindication for Magnetic Resonance Imaging (MRI) assessment; and (7) foreseeable absence of more than 2 weeks during the intervention period. For details of the number of subjects excluded due to the eligibility criteria, the reader is kindly referred to the corresponding publications (Kemmler W. et al., 2014; Wittke et al., 2017). Finally, 120 (PUSH) and 81 men (RUSH) respectively, eligible and willing to participate, were randomly assigned to three (PUSH) or two (RUSH) subgroups with different intervention protocols vs. a non-training control group. However, in this retrospective comparative analysis we focus on overweight-obese (BMI 25–35 kg/m^2^), fully employed (occupational working time: ≥38.5 h/week) men who conducted the HIIT or HIT-RT protocol. Thus, finally 27 participants of the PUSH HIT-RT and 30 participants of the RUSH HIIT intervention groups were included in the present analysis. In order to give the reader an overview of the study-arm effects, we have also listed the results of the 42 eligible (see criteria above) participants of the pooled control group who were not included in the statistical analysis.

### Study Procedure, Intervention

Subjects were thoroughly informed about the does and don'ts by the principal study investigators. This included avoiding intense physical activity and exercise 48 h pre-assessment.

#### Intervention

Apart from introduction, briefing and early conditioning, HIIT/HIT-RT core interventions of both RUSH and PUSH were 16 weeks. For details of study invention the reader is kindly referred to the corresponding publication (Kemmler W. et al., 2014; Wittke et al., 2017; Tuttor et al., 2018).

##### Rush protocol

Briefly, the RUSH study provided a non-linearly periodized 16-week high intensity running protocol that started with 2 sessions per week and progressively increased to 3–4 sessions per week after week 8. At least two of the sessions were performed on a Finnenbahn wood-chip trail. The participants were provided with training logs that set out the intensity, volume and frequency of running exercise for 4 weeks each. Individual prescription of running intensity based on stepwise treadmill tests to a voluntary maximum. Using the “Schwelle” software (Wassermann, 2005), the individual aerobic threshold (IAT) concept of Dickhuth et al. (1991) and Tuttor et al. (2018) (IAT: minimum lactate + 2 mmol/l) was applied to determine the IAT and the heart rate at IAT (IAT-HR), respectively. Of importance, validity of the calculated IAT-HR was tested at baseline and after 8 weeks by a 30 min run at the IAT-HR. The IAT-HR was then adjusted as necessary based on the subjects' perceived exertion and lactate tests. Heart rate watches (Polar RS 400, Kempele, Finland) enabled participants to properly monitor their prescribed heart rate. Depending on the length of the intervals (90 s−12 min), exercise intensity during the HIIT-sessions ranged between 95 and >110% IAT-HR. Rest periods between the high intensity cycles averaged 1–3 min at ≈70–75 IAT-HR and consisted of slow jogging and/or fast walking. Apart from HIT intervals, high intensity continuous running was applied for 25–45 min at the IAT (i.e., 100% IAT-HR) every 4–5th session. Total volume per session including warm up and cool down averaged 40–50 min/session. Two of the three to four sessions/week were consistently supervised by the principal investigator (MT).

##### Push protocol

The PUSH study provided a periodized 16-week high intensity resistance exercise training protocol with 2 to (every 3rd−4th week) 3 sessions/week. All the main muscle groups were addressed by 10–12 exercises/session (from an exercise pool of 17 exercises) using resistance training machines (MedX, Ocala, Fl., USA). Following recent definitions (Gießing, 2008; Giessing et al., 2016), HIT-RT was set as a single set RT to muscular failure+ (Steele et al., 2017a) using intensifying strategies. As with RUSH, participants were provided with 4 week training logs (linearly periodized with each 4th week a recreational exercise week) that prescribed the order of exercises, number of repetitions, intensity (Steele et al., 2017a) and movement velocity (time under tension (TUT in s) during the concentric, isometric, eccentric phase). The number of repetitions was steadily decreased from 8–10 to 3–5 reps over all four 4 weeks phases. After the 4 weeks of this exercise protocol, phase 2 also focused on work to muscular failure (MMF) with rest periods that progressively decreased from 2–3 min to 1 min of rest between the exercises using a TUT of 2 s-1 s-2 s. Phase 3 introduced a superset/compound/giant set strategy with one session per week prescribing a synergistic approach (consecutively blocks of 2–4 exercises for the same muscle group) and one session applying an antagonistic approach (blocks with one exercise each for the agonist and one exercise each for the antagonist consecutively). Rest periods were 1 min within the blocks and 2 min between the blocks. TUT varied between “explosive” -1 s -2 s (range 8–10 reps) and 3 s-1 s-3 s (range <8 reps). During phase 4, we enhanced the muscle effort (MMF+) by prescribing further reps with reduced loads immediately after the initial work to MMF (“drop sets”) using one (week 13–14) or two (week 15–16) drop sets while reducing the load by about 10% each.

Apart from exercise training parameters, training logs completed by the participants asked for rate of perceived exertion/session and net time for conducting the exercise protocol. Attendance and compliance (i.e., proper completion of prescribed length and intensity (IAT-HR) of the exercise bout) of the exercise were monitored by the instructors during the two joint sessions. Of note, HR was not monitored during the HIT-RT sessions of PUSH. Attendance and compliance of the 1–2 non-supervised RUSH sessions were protocoled in the training logs and randomly checked using the memory function of the heart rate watches. Attendance by the PUSH participants was monitored using the chip card system of the gym (Kieser, Erlangen, Germany); compliance with the exercise protocol was checked by instructors who supervised each of the HIT-RT sessions.

### Study Outcome

#### Primary Study Endpoint

Changes in the metabolic syndrome (MetS) Z-Score according to the International Diabetes Federation (IDF; Alberti et al., 2006) from baseline to follow-up (FU).

#### Secondary Study Endpoints

- Changes in left ventricular (LV) stroke volume index as determined by cardiovascular MRI from baseline to FU.
- Changes in LV myocardial mass index at end-diastole as determined by CMRI from baseline to FU.
- Changes in body fat rate from baseline to FU.
- Changes in soft lean body mass from baseline to FU.

#### Explanatory Study Endpoints

- Changes in parameters constituting the MetS according to International Diabetes Federation (IDF; Alberti et al., 2006) from baseline to FU.
  - ° Resting glucose.
  - ° Triglycerides.
  - ° HDL-cholesterol.
  - ° Mean arterial pressure.
  - ° Waist circumference.

### Measurements

Each participant was tested by the same experienced researcher at baseline and follow-up at about the same time of the day (±1 h). FU tests were conducted in the week (i.e., 5–7 days) after the last exercise session.

#### Anthropometry

Height was measured with a stadiometer (Holtain, Crymych Dyfed., Great Britain), body mass and -composition were determined via direct-segmental, multi-frequency Bio-Impedance Analysis (DSM-BIA; Inbody 770, Seoul, Korea). The latter device measures impedance of the trunk, arms and legs separately using a tetrapolar eight-point tactile electrode system that applies six frequencies (1, 5, 50, 250, 500 and 1000 kHz). In order to standardize the test procedure, participants were requested to refrain from intense physical activity 12 h and from nutritional intake 3 h prior to the DSM-BIA assessment. Waist circumference was determined as the minimum circumference between the distal end of the rib cage and the top of the iliac crest along the midaxillary line. Body mass index was calculated body mass (kg)/body height (m^2^).

#### Metabolic Syndrome

The MetS Z-Score was calculated according to the calculation proposed by Johnson et al. (2007), albeit based on the more recent MetS definition presented by the IDF (Alberti et al., 2006) instead of the NCEP-ATP-III definition (Expert-Panel, 2001). Using this approach, MetS is prevalent if waist circumference is increased (≥94 cm for Caucasian males) and two of the following four factors are also present: (1) reduced HDL-C (<40 mg/dl for males; or specific treatment for reduced HDL-C); (2) raised triglyceride (TriGly) levels ≥ 150 mg/dl (or specific treatment); (3) raised blood pressure (≥85 or ≥135 mmHG, or specific treatment); (4) raised fasting plasma glucose (≥100 mg/dl, or previously diagnosed type 2 diabetes). Based on these cut-off points, the individual participant data and the corresponding baseline standard deviation (SD) of the entire cohort the Z-Score were calculated as follows: [(40 –HDL-cholesterol)/SD HDL-C] + [(triglycerides – 150)/SD TriGly] + [(Glucose – 100)/SD Glucose] + [(waist circumference – 94)/SD WC] + [(Mean arterial (blood) pressure (MAP) – 107.5)/SD MAP].

Blood pressure was determined in a sitting position after 5 min rest with an automatic oscillometric device (Bosco, Bosch, Jungingen, Germany). Subjects were requested to avoid intense physical activity 12 h prior to the assessment and to refrain from coffee or tea for at least 3 h prior to testing.

After an overnight fast, blood was sampled in the morning (7:00 a.m. to 9:00 a.m.) in a sitting position from an antecubital vein. Serum samples were centrifuged at 3000 RPM for 20 min and immediately analyzed by the Medical Department of the FAU. Glucose, total cholesterol, HDL-and LDL-cholesterol and triglycerides (Olympus Diagnostica GmbH, Hamburg, Germany) were determined.

For details of the CMRI procedure and image analysis the reader is kindly referred to other publications (Scharf et al., 2015, 2017). Briefly, the CMRI of both studies were consistently conducted on a 1.5 Tesla device (Magnetom Avanto, Siemens Erlangen, Germany) using a six-channel phased array surface and spine matric receiver coil. Four-, three- and two-chamber long- and short-axis cine images were compiled while using breath-hold balanced steady-state free-precession sequences with retrospective electrocardiographic gating. The following scan parameters were applied: field of view: 215 to 265 × 300 to 340 mm^2^; slice thickness: 6 mm; intersection gap: 1.5 mm; repetition/echo time: 41.25 to 50.7/1.12 to 1.38 ms; flip angle: 61° to 75°; pixel size: 1.5 to 2.8 × 1.2 to 2.0 mm^2^; matrix: 105 to 156 × 192 to 256; number of reconstructed phases: 25; integrated parallel acquisition techniques (PAT) acceleration factor: 2.

#### CMR Image Analysis

Quantitative image analysis of both studies were consistently performed using Argus 4.01 software (Siemens, Erlangen, Germany). Left (LV) and right ventricular (RV) functional analysis was independently performed by two experienced researchers. Tracing of the endo- and epicardial borders from base to apex was conducted manually at end-diastole and -systole. Papillary muscles and epicardial adipose tissue were excluded from the analysis. Stroke volume was calculated as end-diastolic volume—end-systolic volume. The myocardial mass of the left ventricle was measured at end diastole by multiplying the myocardial volume by the specific gravity of myocardium (1.05 g/ml). All results were divided by body surface area (BSA) to adjust data for weight and height.

Baseline characteristics and confounding factors (i.e., lifestyle, diseases, medication, physical activity, exercise) were assessed at baseline and FU by standardized questionnaires and personal interviews. The participants' dietary intake was assessed pre- and post-trial by a 4-day dietary protocol conducted by all participants. The consumed food was analyzed using the Freiburger Ernährungs-Protokoll [Freiburger Nutrition Protocol] (nutri-science, Hausach, Germany).

### Changes in Trial Outcomes After Trial Commencement

No changes of trial outcomes were made after trial commencement.

### Sample Size Analysis

Focusing on differences between HIIT and HIT-RT for the primary study endpoint “MetS Z-Score,” we expected higher reductions of MetS Z-Score in the HIIT compared with the HIT-RT protocol. Based on a MetS Z-Score reduction of −2.06 ± 1.31 in the HIIT (Kemmler W. et al., 2014) and −1.03 ± 1.56 in the HIT-RT (Kemmler et al., 2016c) for the entire corresponding study arm, 31 participants per groups were needed to verify a α = 0.05 with 80% power. However, since we expected more pronounced differences for overweight to obese people with a corresponding higher risks or prevalence of the MetS, we decided to conduct this analysis with a slightly lower sample size.

### Randomization Procedures and Enrollment

Stratified for age (5-year strata), 81 (RUSH) and 120 (PUSH) participants were randomly assigned to two (RUSH) or three (PUSH) study arms: (a) HIIT or HIT-RT; (b) waiting-control group (CG) and for PUSH only (c) HIT and protein supplementation using a uniform allocation rate (1:1 or 1:1:1). However, the randomization methods differ between the studies. While RUSH used a computer-generated random list provided by an independent statistician to allocate participants to the study groups (i.e., allocation sequence generation), in the PUSH study lots were drawn by the participants themselves. Lots were put in opaque plastic shells (“kinder egg,” Ferrero, Italy), and drawn from 4 bowls with three lots each (HIT-RT, CG; HIT-RT&Protein) in order to generate strata of 5 years and a uniform allocation rate. Independently of the randomization strategy, neither participants nor researchers knew the allocation beforehand. Subsequently, status of the participants was listed by the primary investigator (MT) who enrolled participants and instructed them in detail about their status including corresponding dos and don'ts.

### Blinding

While participants and instructors were aware of the group status, research assistants were kept blind to the allocation of the participants and were not allowed to ask, either.

### Statistical Analysis

An intention to treat analysis was applied that included all the participants who were randomly assigned independently of lost to follow-up or compliance. R statistics software was used in combination with multiple imputation by Amelia II. The full data set was used for multiple imputation, with imputation being repeated 100 times. Over-imputation diagnostic plots provided by Amelia II confirmed that the multiple imputation worked well in all cases. Based on a statistically and graphically checked normal distribution of the primary and secondary outcomes presented here, dependent *t*-tests were used to analyze within-group changes. Due to our hypothesis, we consistently focused on the two group comparison of HIIT and HIT-RT in our statistical analysis. Corresponding group differences between the exercise groups vs. control group were not addressed. Thus, Welch *t*-Tests were used to analyze differences between HIIT and HIT-RT for all primary and secondary study endpoints. However, only in order to allow the reader to assess the net changes in the exercise groups, changes in the control groups were additionally listed in **Tables 2–5**. All tests were 2-tailed, significance was accepted at *p* < 0.05 or adjusted *p* < 0.05, respectively. Effect sizes (i.e., standardized mean differences) between HIIT and HIT-RT for primary and secondary study outcomes were calculated using Cohen's d (Cohen, 1988). Effect sizes below d' < 0.20 were considered negligible, 0.2− <0.5 as low, 0.5− <0.8 as moderate, 0.8− <1.3 as large and d'≥1.3 as very large. Apart from R-statistic software and Amelia II, all the other statistical procedures were performed with SPSS 25 (SPSS Inc., Chicago, IL, USA).

## Results

### Baseline Characteristics, Participant Flow, Lost to Follow-Up, Attendance

Table 1 shows baseline characteristics of the HIT-RT and HIIT groups. In summary, no significant differences between the HIIT and HIT-RT study-arms were observed. Further, data of the CG did not differ relevantly from results of the intervention groups. At baseline, 63% of the participants of the HIIT and 53% men of the HIT-RT were diagnosed as having the MetS according to IDF (Alberti et al., 2006).

**Table 1:** Baseline data for the HIT-RT and HIIT study with between group differences.

**Variable**		**HIT-RT (*n* = 27) MV ± SD**	**HIIT-END (*n* = 30) MV ± SD**	***P*-value**
Age [years]		43.1 ± 5.6	43.6 ± 5.0	0.748
Body height [cm]		180.0 ± 6.9	180.3 ±7.5	0.807
Body mass [kg]		92.3 ± 12.0	93.8 ±12.8	0.663
BMI [kg/m^2^]		28.5 ± 2.6	28.9 ± 2.4	0.692
Occupational working time [h]^a^		43.9 ± 3.7	44.7 ± 3.1	0.410
Physical activity [Index]^b^		2.93 ± 1.43	2.90 ± 1.38	0.592
Training volume [min/week]^a^		28 ± 31	29 ± 32	0.918
Energy Intake [kcal/d]^c^		2614 ± 763	2697 ± 762	0.894
Protein Intake [g/kg/d]^c^		1.07 ± 0.50	1.18 ± 0.54	0.416
CHO/Fat/Alcohol [g/d]^c^		285/108/13	307/98/15	>0.403
Hypertonia [n]^a^		4	4	0.872
Diabetes [n]^a^		2	1	0.492
Antihypertensive drugs [n]^a^		3	4	0.799
Smoker [n]^a^		2	3	0.730
Ventricular ejection fraction [%]	LV	62.1 ± 7.1	61.1 ± 6.8	0.709
	RV	61.1 ± 7.0	60.9 ± 7.3	0.581

*Data of the control group are shown (right column), but not included in the inference-statistical analysis listed in the table. MV, mean value; SD, standard deviation*.

a *As assessed by baseline questionnaires*.

b *Based on a scale from 1 (very low) to 7 (very high) according to a subjective assessment of professional, household, and recreational activities*.

c *Based on a 4-day dietary intake protocol*.

While no participant of the HIT-RT withdrew, four subjects of the present HIIT group were lost to follow-up. Two of these men reported running-related complaints as a reason for their withdrawal. The attendance rate was significantly higher (*p* < 0.001) in the HIT-RT (93 ± 5%) compared with the HIIT group (83 ± 8%); however, due to the higher training frequency, net exercise attendance (HIIT: 41 ± 5 sessions vs. HIT-RT: 36 ± 3 sessions) was higher (*p* < 0.001) in the HIIT. Average exercise time/session was 37 ± 3 min (including 3–5 min warm up) in the HIT-RT vs. 50 ± 4 min/session (including 10 warm-up and 5 cool-down) in the HIIT (*p* = 0.001). Apart from periods of muscle pain and delayed onset of muscular soreness (DOMS), no further exercise-induced complaints were reported in the HIT-RT. In contrast, about one third of the HIIT group reported frequent periods of hip, knee or (rarely) ankle problems related to the running exercise; as mentioned, two participants quit the study due to joint problems.

### Primary and Secondary Outcomes

Table 2 shows data for primary and secondary study endpoints. In order to allow the reader to adequately estimate changes in the exercise groups we additionally provide changes in the CG. Based on comparable baseline values, in summary we confirmed our primary hypothesis that (1) HIIT is significantly (*p* = 0.049, d' = 0.54) more effective for favorably affecting the Metabolic Syndrome Z-score (MetS-Z-Score) compared with HIT-RT. Additionally, we confirmed our hypothesis that both exercise protocols favorably affect (*p* < 0.001) the MetS Z-Score.

**Table 2:** Baseline data and changes in the Metabolic Syndrome Z-Sore (Met-S-Z-Score) in HIT-RT and HIIT with corresponding between group differences.

	**HIT-RT (*n* = 27) MV ± SD**	**HIIT-END (*n* = 30) MV ± SD**	***P*- value**	**CG (*n* = 42) MV ± SD**
**Metabolic Syndrome Z-score**				
Baseline	0.68 ± 2.26	0.77 ± 2.54	0.896	−0.40 ± 2.55
Changes	−1.28 ± 1.32^***^	−2.01 ± 1.37^***^	0.049	0.09 ± 1.34

*Data of the control group (CG) are shown (right column), but not included in the analysis. MV, mean value; SD, standard deviation*.

*** *p < 0.001*.

Table 3 gives results of our secondary study outcomes. LV stroke volume index and LV myocardial mass index increased significantly (HIT-RT: *p* = 0.001 and *p* ≤ 0.024; HIIT: both *p* < 0.001) in both groups, however in line with our expectation, changes were significantly higher in the HIIT compared with the HIT-RT group (*p* ≤ 0.002); effect sizes for corresponding differences were large – very large (d' = 85 and d' = 1.56). Thus, we confirmed our core secondary hypothesis that HIIT is significantly more effective for favorably affecting (1) ventricular stroke volume index and (2) myocardial mass index compared with HIT-RT.

**Table 3:** Baseline data and changes on core secondary endpoints in the HIT-RT and HIIT with corresponding between group differences.

	**HIT-RT (*n* = 27) MV ± SD**	**HIIT-END (*n* = 30) MV ± SD**	***P*- value**	**CG (*n* = 42) MV ± SD**
**Left ventricular (LV) stroke volume index [ml/m**^**2**^**]**				
Baseline	48.1 ± 7.6	45.4 ± 5.8	0.137	48.3 ± 6.2
Changes	2.18 ± 3.10^***^	5.03 ± 3.60^***^	0.002	0.24 ± 2.07
**LV myocardial mass index at end diastole [g/m**^**2**^**]**				
Baseline	56.6 ± 5.7	58.3 ± 5.9	0.273	55.7 ± 6.3
Changes	1.24 ± 2.76^*^	5.78 ± 3.04^***^	<0.001	−0.37 ± 0.68

*MV, mean value; SD, standard deviation. Data of the CG are shown (right column), but not included in the analysis*.

* p < 0.05;

*** *p < 0.001*.

The body fat rate (Table 4) decreased significantly in both exercise groups (<0.026). In contrast to our expectation, no significant differences were observed between the groups (*p* = 0.498; d' = 0.18). In parallel, changes of LBM (Table 4) did not differ significantly between the groups (*p* = 0.673, d' = 0.11). Of interest, no significant changes (*p* ≥ 0.22) of LBM were observed in the exercise groups. Thus, we have to reject our secondary hypothesis that (1) HIIT is significantly more effective for favorably affecting body fat rate compared with HIT-RT while (2) HIT-RT is significantly more effective for favorably affecting Lean Body Mass (LBM) compared with HIIT.

**Table 4:** Baseline data and changes on secondary endpoints in the HIT-RT and HIIT with corresponding between group differences.

	**HIT-RT (*n* = 27) MV ± SD**	**HIIT-END (*n* = 30) MV ± SD**	***P*- value**	**CG (*n* = 42) MV ± SD**
**Body fat rate [%]**				
Baseline	25.2 ± 3.2	26.6 ± 3.9	0.117	26.6 ± 3.0
Changes	−1.23 ± 1.82^***^	−0.88 ± 2.11^*^	0.498	0.48 ± 1.50
**Soft lean body mass [kg]**				
Baseline	68.8 ± 7.2	68.4 ± 8.0	0.835	68.9 ± 6.8
Changes	0.36 ± 1.66 ^n.s.^	0.15 ± 2.14 ^n.s.^	0.673	−0.32 ± 1.42

Data of the CG are shown, but not included in the analysis. MV, mean value; SD, standard deviation. ^n.s.^ p ≥ 0.05;

* p < 0.05;

*** *p < 0.001*.

Table 5 shows selected parameters constituting the MetS according to IDF (Alberti et al., 2006). Although non-significant for resting glucose (−3.3 ± 11.2 mg/dl, *p* = 0.160), HIIT favorably affects (*p* < 0.001) all MetS components, while HIT-RT significantly improves two out of five components (glucose: −3.1 ± 10.9 mg/dl, *p* = 0.146) (Table 5). However, significant differences between the groups were determined for HDL-C only.

**Table 5:** Baseline data and changes of explorative study outcomes in the HIT-RT and HIIT with corresponding between group differences.

	**HIT-RT (*n* = 27) MV ± SD**	**HIIT-END (*n* = 30) MV ± SD**	***P*-value**	**CG (*n* = 42) MV ± SD**
**Waist circumference [cm]**				
Baseline	103.7 ± 8.3	102.7 ± 7.5	0.615	103.5 ± 7.0
Changes	−2.21 ± 2.63^***^	−2.78 ± 3.06^***^	0.449	0.61 ± 2.29
**MAP**				
Baseline	102.9 ± 9.3	103.4 ± 8.2	0.837	98.3 ± 9.3
Changes	−5.30 ± 5.01^***^	−4.10 ± 5.44^***^	0.376	0.12 ± 3.96
**HDL-C [mg/dl]**				
Baseline	46.9 ± 8.5	42.7 ± 10.7	0.104	47.9 ± 10.9
Changes	1.52 ± 5.06 ^n.s.^	8.33 ± 5.79^***^	<0.001	0.74 ± 5.43
**Triglycerides [mg/dl]**				
Baseline	185 ± 77	179 ± 89	0.104	155 ± 73
Changes	−12.0 ± 35.0 ^n.s.^	−23.1 ± 23.4^***^	0.191	−5.0 ± 36.3

Data of the CG are shown, but not included in the analysis. MV, mean value; SD, standard deviation. ^n.s.^p ≥ 0.05;

*** *p < 0.001*.

### Confounding Variables

Although strong emphasis was placed on the maintenance of lifestyle, diet and exercise, energy intake decreased significantly (−69 ± 176 kcal, *p* = 0.016) in the HIT-RT and increased slightly in the HIIT (29 ± 245 kcal, *p* = 0.701; *p* = 0.123 to HIT-RT). However, protein intake did not change relevantly in the groups (HIT-RT: −2.5 ± 14.6 vs. HIIT 1.1 ± 12.7 g/d, *p* = 0.508). Physical activity and exercise did not change (*p* ≥ 0.601) according to the FU questionnaires, however after one-on-one interviews with participants with conspicuous results for body composition changes, two participants each of the HIT-RT and CG group admitted to starting endurance exercise training (1.5–2.5 sessions of 30–60 min/w.) and/or (HIT: *n* = 3, CG: *n* = 1) reduced energy consumption (≈10–20%). No participant of the HIIT group reported corresponding changes of confounding parameters. Apart from these changes in lifestyle, no changes of medication or incidence of new diseases were reported by the participants of the groups.

## Discussion

The aim of this study was to determine the comparative effect of resistance vs. endurance exercise on cardiometabolic parameters in overweight-obese men using the time-effective high intensity training method. HIT-RT is defined as single set resistance exercise training to muscular failure using intensifying strategies (Gießing, 2008; Steele et al., 2017a) and HIIT is defined as repeated very short (<45 s) or short (2–4 min) bouts of high to near maximum intensity exercise (Buchheit and Laursen, 2013). Due to their low exercise volume and corresponding time effectiveness, HIT protocols might be a particularly suitable exercise strategy for middle-aged, fully employed men (Rommel et al., 2008), a group with low time resources.

Several studies that focus on HIIT and the few studies that evaluate HIT-RT protocols showed that they are effective for favorably affecting cardiometabolic risk factors and functional cardiac parameters (Gibala, 2007; Haykowsky et al., 2013; Kemmler W. et al., 2014; Scharf et al., 2015, 2017; Kemmler et al., 2016a,c; Batacan et al., 2017; Wilson et al., 2019) in different male cohorts. In general, our study confirmed these results for a cohort of overweight-obese middle-aged men, however the aim of the present study was not “proof of principle” but to compare HIT endurance and resistance protocols with respect to their dedicated effect on cardiometabolic and cardiac parameters.

In summary, our results indicate that a HIIT (endurance) protocol was superior to HIT–RT program for improving the Metabolic Syndrome Z-Sore (*p* = 0.049) and developing parameters of cardiac morphology and performance (*p* < 0.001). Surprisingly, no significant difference was determined for LBM and body fat rate. Further, reviewing the components of the Metabolic Syndrome, apart from HDL-C with more favorable changes in the HIIT (*p* < 0.001), changes of waist circumference, MAP, resting glucose and triglycerides improved favorably in both groups to a similar high extent.

Only few exercise trials (Banz et al., 2003; Stensvold et al., 2010; Bateman et al., 2011; Earnest et al., 2014; Sigal et al., 2014) focus on the direct comparison of resistance (RT) vs. aerobic (endurance) training (AET) with respect to cardiometabolic and cardiac markers. To our best knowledge, however, apart from the protocol (Ramirez-Velez et al., 2016) of an otherwise unpublished study, none of them focus on HIT-strategies for AET and RT. Revisiting the MetS, results of studies comparing AET and RT were quite heterogeneous. Bateman et al. (2011) and Earnest et al. (2014) reported significant differences for the MetS-Score in favor of AET with no or minor effects of RT on the MetS. In contrast, Sigal et al. (2014) and Stensvold et al. (2010) observed comparable favorable effects of endurance and resistance exercise on MetS components. In line with the present study, the two latter studies (Stensvold et al., 2010; Sigal et al., 2014) reported favorable changes of LBM and body fat rate in their endurance and resistance study arms without significant differences between the groups. Banz et al. (2003), who focus on CAD risk factors in a small cohort of middle aged-older overweight men, listed significantly higher reductions of body fat rate in their RT compared with their AET group. The latter study (Banz et al., 2003) further supports our result of high HDL-C increases after aerobic exercise while the effect of RT was also negligible. Thus, although both AET and RT protocols are generally effective for positively impacting the metabolic syndrome, some components of the METS differ considerably in their adaptive response. As a consequence, summarizing RT and AET is not appropriate for evaluating the effect of “exercise” on cardiometabolic health (Lin et al., 2015). Apart from differences in exercise type, the intensity and volume of the particular exercise protocol are relevant predictors of cardiometabolic effects. While there is an ongoing discussion whether HIIT or MICE (moderate intensity continuous exercise) endurance protocols are more effective for impacting the MetS (e.g., Johnson et al., 2007; Tjonna et al., 2008; Earnest et al., 2013; Kemmler W. et al., 2014; Ramirez-Velez et al., 2017) and related anthropometric or cardiometabolic parameters (review in e.g., Hansen et al., 2010; Hwang et al., 2011; Weston et al., 2013; Wewege et al., 2017; Costa et al., 2018; Andreato et al., 2019), the effect on cardiac parameters^1^ is much more pronounced after HIIT protocols (e.g., Scharf et al., 2015; Huang et al., 2019). Unfortunately, corresponding data on HIT-RT (vs. high volume, low intensity RT) are not available. Spence et al. (2011) who applied CMRI to monitor ventricular adaptation from 24 weeks of endurance (*n* = 10) or resistance exercise (*n* = 13) reported significant favorable changes of morphometric and functional CMRI parameters in the endurance group only, while there were positive, albeit non-significant, effects in the RT. Apart from the (too) low statistical power, another difference between the present study and the study of Spence et al. (2011) is the more intense HIIT or HIT-RT intervention. More recently, Christensen et al. (2019) reported a comparable effect on left ventricular mass from endurance and resistance training. Of note, the authors also observed a significant reduction of epicardial adipose tissue mass after endurance and resistance exercise (32 and 24% respectively). However, while the effect on pericardial adipose tissue mass after endurance training failed to reach statistical significance, resistance training significantly reduced pericardial adipose tissue mass by 31% (*p* < 0.001). Thus, the nimbus of superiority of endurance exercise protocols in the area of cardiac health is not justified.

However, in a recent study we observed similar results of HIT-RT (as defined as single set RT to muscular failure) and high intensity multiple set RT (also to muscular failure) on the cardiometabolic syndrome Z-Score (Kemmler et al., 2016c). Thus, in parallel to AET (Swain and Franklin, 2006) there is some evidence that intensity rather than volume (per session) of RT might be the critical exercise parameter for triggering cardiometabolic effects.

Revisiting the practical application of our finding, an argument frequently cited for the implementation of HIIT and HIT-RT protocols in public health settings are their time efficiency. We confirmed this aspect fully for HIT-RT whole body exercise protocols with their net exercise time of below 35 min/session (Kemmler et al., 2016b; Wittke et al., 2017). Less clearly, HIIT protocols applied in health care settings vary considerably in interval (30 s to 4 min) and rest period (60 s to 3 min) length (Weston et al., 2013; Wewege et al., 2017; Costa et al., 2018). Further and in contrast to MICE, the high musculoskeletal strain entailed by HIIT may well make complex, and ultimately time consuming, warm-up protocols inevitable in order to prevent muscle damage and injuries. Nevertheless, in a recent systematic review and meta-analysis of HIIT vs. MICE effects in overweight to obese adults, Wewege et al. (2017) reported a significantly lower training time (i.e., ≈29 min^2^ vs. ≈42 min/session) when applying HIIT.

At this point, we would like to draw the reader's attention to some limitations and features of this study. (1) The main study feature—which can be also considered as a study limitation however—was that we addressed the comparison between HIIT and HIT-RT, not in a parallel group design but consecutively in two trials. In actual fact, the project should be regarded as a combination of two randomized controlled trials with identical eligibility criteria, sample size/group, assessments, statistical procedures, and comparable length of the intervention. Nevertheless, from a methodological point of view this approach is problematic and might confound important aspects of our study. A less prominent problem might be the time effect. HIIT ran from September to December, HIT-RT was conducted between January to May. Thus, season changes of physical activity or diet may have impacted our results, although no corresponding changes were detected from the FU questionnaires. More importantly, participants were randomly assigned to an exercise or control group, but randomization did not address group allocation to HIT-RT or HIIT, which is the main issue of this contribution. Due to this inadequate randomization and stratification approach, it might have been possible that resultant differences affected our results. However, as listed in Tables 1–5, we did not observe corresponding group differences for baseline characteristics. (2) In the present contribution we exclusively focus on differences between HIT-RT and HIIT; the CG was not included in the analysis in order to prevent problems related to multiple testing. Corresponding data was released in previous publications (Kemmler W. et al., 2014; Kemmler W. M. T. et al., 2014; Scharf et al., 2015, 2017; Kemmler et al., 2016c; Wittke et al., 2017). However, in order to allow the reader to estimate the dimensions of changes in the HIIT and HIT-RT, we added the results of the CG in Tables 2–5. (3) With intervals of 90 s to 12 min but (rarely applied) also continuous bouts (25–40 min) at the IAT, our HIIT approach differs from purebred “HIIT” protocols defined as repeated very short (<45 s) or short (2–4 min) bouts of high to near maximum intensity exercise (Buchheit and Laursen, 2013). (4) One may also criticize the less rigorous control of the exercise protocol at least in the HIIT-group. Indeed, only 2 out of 3–4 sessions were supervised; in addition, we did not consistently monitor heart rate watches in order to check whether participants actually conducted their individual sessions. Further, with respect to compliance with exercise intensity we did not analyze all the heart rate watches after the session, but randomly selected 15–20 participants (i.e., 50%) for this procedure. On the other hand, after monitoring the training logs of the HIT-RT and comparing the rate of repetitions to load we are not always convinced whether participants really worked to MMF. However, considering the close and sincere communication between researchers and participants we conclude that participants closely adhered to the exercise protocol. (4) Contrary to the commitment given, 5 HIT-RT participants started relevant endurance exercise and/or energy reduction programs. Excluding these subjects from the analysis resulted in slightly higher LBM changes (0.45 vs. 0.36 kg, Table 4) but lower body fat reductions (1.18 vs. 1.32 kg, Table 4), and did not relevantly confound our results of non-significant group differences. (5) We opted to use the MetS-Z-Score, a single continuous score based on individual participant data and cut off values for MetS criteria (Johnson et al., 2007). However, more recognized cardiometabolic parameters might have increased the evidence and generalization of the study. (6) We put together a homogeneous cohort of untrained middle-aged men for whom the relevance of time-efficient exercise protocols might be of particularly interest. With respect to generalizability, one may argue that exhausting HIT approaches might be limited to motivated or predominately healthy younger cohorts. However, considering that (a) HIIT was reported to be perceived more “enjoyable” compared with the monotone MICE (Bartlett et al., 2011) and (b) HIIT and HIT-RT protocols were applied in cardiac rehabilitation (e.g., Haykowsky et al., 2013) or with older cohorts (e.g., Steele et al., 2017b), we do not support the latter limitation. (7) In summary, there is a considerable amount of evidence that in parallel to combined resistance and endurance exercise (e.g., Bakker et al., 2017), the effect of combined HIIT and HIT-RT protocols might result in more pronounced effects. This still has to be proven, however, considering the premise of time efficiency.

## Conclusion

In this contribution we determined positive effects of HIIT or HIT-RT on cardiometabolic, cardiac and morphometric parameters closely related to cardiometabolic health. However, at least for the outcomes addressed here, HIIT effects were on average more pronounced. Nevertheless, we conclude that overweight to obese people can freely choose their preferred exercise type (AET or RT) to positively affect their cardiometabolic risk, while investing an amount of time that should be feasible for everybody.

## Data Availability Statement

The datasets generated for this study are available on request to the corresponding author.

## Ethics Statement

The studies involving human participants were reviewed and approved by Ethics committee of the Friedrich Alexander University Erlangen-Nürnberg; Krankenhausstrasse 12, 91052 Erlangen. The patients/participants provided their written informed consent to participate in this study.

## Author Contributions

MT, SS, MK, ML, MS, MU, AW, and WK designed the study, completed data analysis and/or interpretation and drafted the manuscript. MT, AW, SS, MS, AW, and WK contributed to study conception and design and revised the manuscript. MT and WK accepts responsibility for the integrity of the data sampling, analysis, and interpretation. All authors contributed to the article and approved the submitted version.

## Conflict of Interest

The authors declare that the research was conducted in the absence of any commercial or financial relationships that could be construed as a potential conflict of interest.

## Footnotes

^1^E.g., left and right ventricle end-diastolic and systolic volume index, stroke volume index, Mass index at end-diastole, myocardial mass (Scharf et al., 2015), left ventricular (LV) contractile and diastolic functions (Huang et al., 2019).

^2^However, warm-up might not be consequently considered when calculating the length of the session.
